# Effectiveness of State and Federal Government Agreements with Major Credit Card and Shipping Companies to Block Illegal Internet Cigarette Sales

**DOI:** 10.1371/journal.pone.0016754

**Published:** 2011-02-14

**Authors:** Kurt M. Ribisl, Rebecca S. Williams, Ziya Gizlice, Amy H. Herring

**Affiliations:** 1 Gillings School of Global Public Health, University of North Carolina, Chapel Hill, North Carolina, United States of America; 2 Lineberger Comprehensive Cancer Center, University of North Carolina, Chapel Hill, North Carolina, United States of America; 3 Center for Health Promotion and Disease Prevention, University of North Carolina, Chapel Hill, North Carolina, United States of America; 4 Carolina Population Center, University of North Carolina, Chapel Hill, North Carolina, United States of America; Yale University School of Medicine, United States of America

## Abstract

Most Internet vendors offer tax-free cigarettes making them cheaper than those sold at stores. This undermines the impact that higher prices have upon reducing consumption. Most Internet tobacco sales have violated taxation and youth access laws, which led to landmark voluntary agreements in 2005 with the major credit card companies and major private shippers to ban payment transactions and shipments for all Internet cigarette sales.

**Objective:**

To assess whether these bans increased the rate of Internet Cigarette Vendors (ICVs) ceasing online sales, decreased the proportion of vendors offering banned payment and shipping options, and decreased consumer traffic to the most popular ICVs.

**Design:**

Websites in a longitudinal study of ICVs were visited in 2003 (n = 338), 2004 (n = 775), 2005 (n = 664), 2006 (n = 762), and 2007 (n = 497) to assess whether they were in business and monitor their advertised sales practices. The number of unique monthly visitors to the 50 most popular ICVs at baseline was examined for the period one year before and two years after the bans to determine whether the bans altered traffic.

**Results:**

Following the bans, the rate of ICVs ceasing online sales year to year increased, but due to an influx of new vendors, there was a net increase in ICVs. The proportion of vendors accepting banned *payment* options dropped from 99.2% to 37.4% after the bans, and the proportion offering banned *shipping options* dropped from 32.2% to 5.6%, but there was a corresponding increase in vendors offering non-banned payment options (e.g., personal checks) and shipping options (e.g., US Postal Service). Following the bans, there was a 3.5 fold decline in traffic to the most popular ICV websites.

**Conclusions:**

This promising approach to controlling the sale of restricted goods online has implications for regulating other products such as alcohol, firearms, quack cures, and medicines sold without a prescription.

## Introduction

The widespread availability of cheap cigarettes online from Internet cigarette vendors (ICVs) is a public health concern for several reasons. First, higher cigarette prices reduce the demand for cigarettes thereby reducing cigarette consumption, smoking prevalence, and youth smoking initiation. [1], [2], [3] However, the availability of cheap and low-tax cigarettes online undermine smokers' quit attempts [4] and undercuts government efforts to reduce consumption through cigarette excise taxes. [5], [6], [7] Second, ICVs' tax avoidance tactics deprive states and the federal government of millions of dollars in annual tax revenue, [8] some of which is used to fund prevention and health care programs. For example, 78% of ICVs advertise on their Website that they sell cigarettes “tax free.” [5] One report [9] estimates that the state of New York lost between $106–122 million in tax revenues in 2004 from Internet sales, and an economist testified at Congressional Hearings [10] that state and local governments in the U.S. lost $552.4 million due to online cigarette sales in Fiscal Year 2003. Third, few ICVs adequately verify buyers' age and identity, and as a result most sell cigarettes to minors when tested. [11], [12]

Because of multiple violations of state and federal laws governing taxation and sales to minors, the U.S. Bureau of Alcohol, Tobacco, Firearms and Explosives and several state Attorneys General (AGs) reached a landmark voluntary agreement on March 17, 2005 with the major credit card companies and PayPal to ban the processing of credit card payments for ICVs. [13] All major private carriers, such as UPS and FedEx, entered into a similar agreement on October 24, 2005 whereby all agreed that they would not deliver cigarettes to consumers, [14] although FedEx [15] had stopped consumer shipments years earlier. States have made several attempts to curtail illegal Internet tobacco sales with limited success, [16] but the credit card and shipping bans were the first serious national attempt to curtail the unlawful behavior of ICVs. Although the policy has a number of impressive features, there are some loopholes that might undermine whether the policy actually reduces Internet cigarette sales. For instance, the payment ban did not apply to mailed personal checks and money orders or to electronic checks submitted online, and the shipping ban did not apply to the United States Postal Service (USPS).

The purpose of this study was to assess the impact of the credit card and shipping bans. We examined three primary outcomes of interest assessed before and after the bans and hypothesized there would be an increase in the rate of vendors ceasing online sales, a decrease in the proportion of vendors offering banned payment and shipping options, and a decrease in consumer traffic to the most frequently visited ICV websites.

## Methods

### Design and Sample

As part of an ongoing longitudinal study [5] of Internet cigarette vendor sales practices started in 1999, [17] cross-sectional samples of ICVs were identified in January 2003, 2004 and 2005, all prior to the bans, and in January 2006, after the bans. The websites of all vendors identified in a given year were revisited the following year to determine if they were still in business, and new vendors were identified with a similar methodology, which is described briefly here and in greater detail elsewhere. [5] Manual searching of online shopping portals and search directories was supplemented with automated searching strategies developed by a private online risk management firm, Cyveillance. Intelligent web spiders reviewed over 40 million websites, nearly 100,000 message boards and newsgroups, and 1 million spam email messages; algorithms were used to winnow this content and identify websites with a high probability of being ICVs. All of these websites were manually visited by trained research assistants to verify the site was English language, was active, and advertised selling cigarettes for remote delivery sale. All websites identified for inclusion in the study were archived with Internet Researcher 2.1 for offline review and content analysis. Screening and archiving of sites was completed by the end of March for all years.

### Content Analysis of ICV Websites

Advertised vendor sales practices were assessed with a customized online data collection tool used to record data from each of the websites in the study sample. Each website was coded by two independent trained raters, and all inter-rater discrepancies were then reviewed and resolved by a more senior project staff member. Outcomes included advertised vendor payment and shipping methods. Payment methods tracked included credit card, money order, personal check, e-check, PayPal, bank/wire transfer, and cash on delivery. Delivery services tracked included USPS, FedEx, UPS, DHL, air mail, and international postal services. This study was deemed exempt by our IRB because it did not have human subjects.

### ICV Traffic Analysis

Longitudinal data indicating the number of unique visitors per month for about two years before and after the bans were obtained for the 50 ICVs that were receiving the most traffic in January 2005, two months prior to the implementation of the credit card payment ban. ComScore, a commercial firm that tracks and estimates website usage and traffic from a panel of 1 million US users, provided 49 months of traffic data (from January 2003 to January 2007). After examining count data and residuals that were 3 standard deviations away from zero, five data points were set to missing.

### Data Analysis: Content Analysis of ICV Websites

Descriptive statistics were calculated with SPSS 16.0, tracking changes in the number of sites and in their sales practices following the bans. We examined changes in the proportion of vendors going out of business, joining the market, and offering banned payment and shipping options for the full sample of ICV websites and for the 50 most popular sites.

### Data Analysis: ICV Traffic Analysis

Although we purchased monthly traffic data dating back to January 2003 for the 50 ICV websites that were most visited in January 2005, many of these websites were not in operation in 2003 or early 2004 yielding lots of missing data. Therefore, we restricted our analyses to March 2004 (12 months prior to credit card ban) to January 2007. Traffic values were set to missing until the site started receiving traffic (63 data points).

ComScore provides a symbol rather than a numeric unique visitor estimate for sites that have only been visited by a few panel members, indicating that the number is non-zero, but an accurate estimate cannot be provided. There were 251 such data points out of 1750 (35 months ×50 sites). Because valid data were obtained in previous and subsequent months (indicating the sites were actively receiving traffic), we imputed these data points depending on whether they were after the credit card ban (n = 230) or before (n = 21). Missing values for a site were replaced with half of the lowest estimated count from that specific site and time period (before or after the credit card ban). For example, 247tobacco.com was missing a unique visitor estimate for June 2005 (after the credit card ban). The missing value was replaced with 715, which was half of the lowest unique visitor estimate for that site after the credit card ban, in December 2005. As a sensitivity analysis, we also ran a model imputing the lowest unique visitor estimate for the 251, and this did not alter the results.

We analyzed the ICV traffic data using a generalized linear mixed growth model allowing random intercepts for each site, and assuming counts follow a negative binomial distribution with a log link (instead of Poisson due to over-dispersion) using SAS Software version 9.2. Furthermore, we adjusted for overall growth in US internet traffic using a log of monthly US internet traffic estimates as an offset variable. First, we fitted a saturated growth model to estimate monthly average internet traffic for the top fifty sites and tested average monthly internet traffic 12 months before the credit card ban and 22 months after the ban to assess the effects of the credit card ban. Similarly, we tested the impact of the shipping ban by contrasting the average internet traffic 6 months before and 15 months after the shipping ban. In addition, we fitted a piecewise linear mixed model with two change points (March 2005 and October 2005) corresponding to the credit card and shipping bans to examine the rates of changes (slopes) in the internet traffic (a) before credit card ban, (b) after the credit card ban but before the shipping ban, and (c) after the shipping ban.

## Results

### Content Analysis of ICV Websites

Websites were identified and visited in 2003 (n = 338), 2004 (n = 775), 2005 (n = 664), 2006 (n = 762), and 2007 (n = 497).

### Vendor attrition

**Figure 1:**
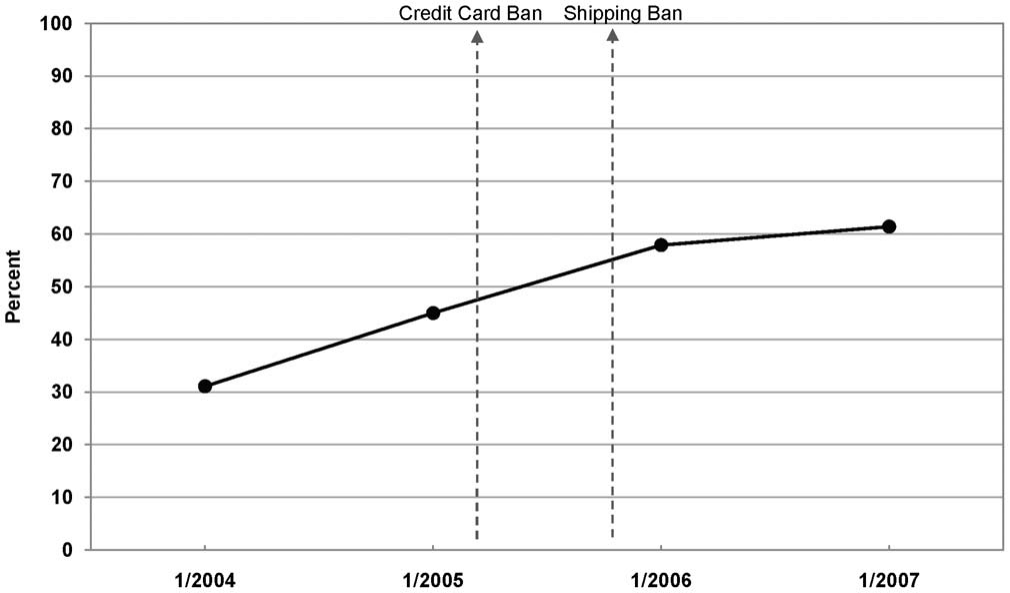
Internet cigarette vendor attrition over time, 2004–2007. Proportion of vendors identified a year earlier whose websites were no longer in operation (1/2006 values represent attrition of sites identified 1/2005, before the bans were implemented).

Prior to the bans, there was a secular increase in vendor attrition. The proportion of vendors identified in January 2003 that were not in business by January 2004 was 31.1% (**Figure 1**). The attrition rate continued to rise over time, such that January 2005, just before the bans, 45.0% of vendors identified a year earlier were no longer in business. The 2005 bans resulted in the highest vendor attrition rates yet: 57.9% of vendors identified in January 2005 were not in business by January 2006, and 61.4% of vendors identified in 2006 were not in business by 2007.

**Figure 2:**
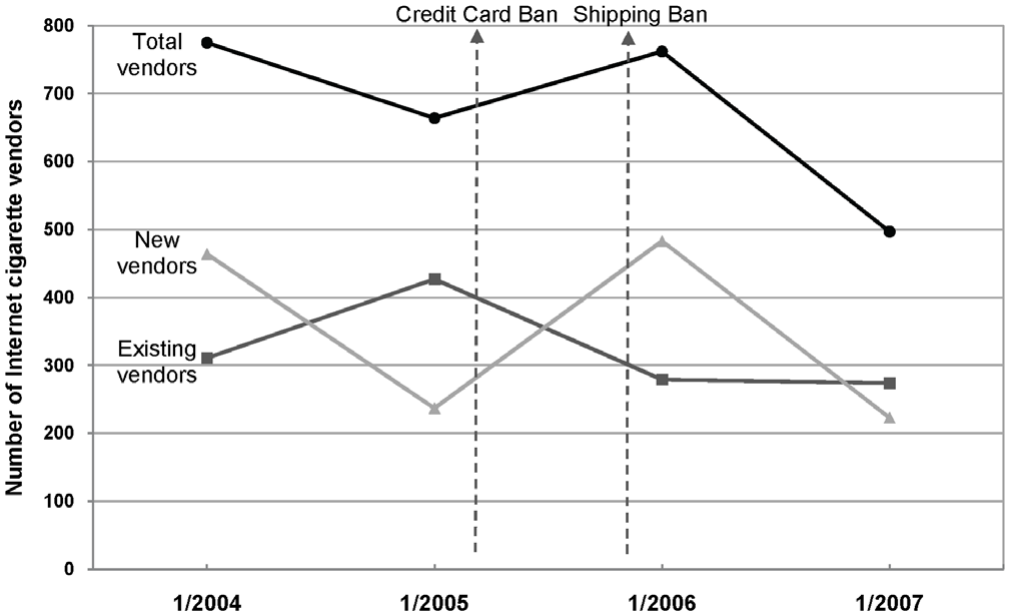
Internet cigarette vendors before and after the credit card and shipping bans. Distribution of new vs. existing Internet cigarette vendors before and after the credit card and shipping bans, 2004–2007.

Despite vendor attrition rising each year, the *total number* of vendors identified rose from 2005 (before the bans) to 2006 (after the bans). **Figure 2** shows that while the number of previously existing vendors dropped from 2005 to 2006, there was a sharp rise in the number of new vendors from 2005 to 2006, indicating that although many vendors went out of business after the bans, many more new vendors entered the marketplace, resulting in 97 more vendors overall in 2006 than in 2005. In the year that followed, while the number of existing sites stayed relatively stagnant, the number of new vendors dropped markedly resulting in an overall drop in the number of vendors.

### Vendors offering banned payment and shipping options

As is described in **Table 1**, the proportion of vendors advertising on their site that they accepted credit cards and PayPal dropped markedly following the credit card ban, but this was countered by an increase in the proportion of vendors accepting checks and money orders. The decline in credit card acceptance was less pronounced for the top 50 ICV sites than for the overall sample. Prior to the bans in 2005, 99.2% of the full sample advertised that they accepted credit cards and PayPal, but after the bans in 2006, only 37.4% did. Among the top 50 vendors, 100% accepted these payment methods before the bans, which only dropped to 61.1% after the bans. In that same time period, the proportion of all ICVs accepting money orders and personal checks rose from 36.4% before the bans to 78.3% after. The percent of the top 50 ICVs accepting checks and money orders rose from 30% before the bans to 80.6% after.

**Table 1:** Advertised payment and shipping practices of Internet cigarette vendors before and after the 2005 credit card and shipping bans.*

	Jan 2004 Pre-Ban # (%)	Jan 2005 Pre-Ban # (%)	Jan 2006 Post-Ban # (%)
Advertised sales practices of top 50 vendors**			
Accept credit cards/PayPal†	37 (100.0)	50 (100.0)	22 (61.1)
Accept check or money order	11 (29.7)	15 (30.0)	29 (80.6)
Ship via UPS/FedEx/DHL†	18 (48.6)	25 (50.0)	5 (13.9)
Ship via USPS	30 (81.1)	45 (90.0)	34 (94.4)
Advertised sales practices of all vendors			
Accept credit cards/PayPal†	771 (99.6)	660 (99.2)	285 (37.4)
Accept check or money order	229 (29.6)	242 (36.4)	597 (78.3)
Ship via UPS/FedEx/DHL†	209 (27.0)	214 (32.2)	43 (5.6)
Ship via USPS††	537 (69.4)	555 (83.5)	706 (92.7)

*The credit card ban was implemented in March 2005 and the shipping ban in November 2005.

**Refers to 50 most popular vendors in January 2005, which were used for the ICV website traffic analysis. 13 of these vendors were new to the sample in January 2005, and by January 2006, 14 of the top 50 vendors had gone out of business.

† Denotes banned payment or shipping methods.

†† The number of vendors shipping via USPS includes vendors shipping via international postal services which would ultimately be delivered in the US by USPS.

A similar pattern can be seen in vendor response to the shipping ban. Right before the ban, 32.2% of all ICVs advertised shipping packages via UPS, FedEx, or DHL, but only 5.6% did so after the bans. Among the top 50 vendors, 50% accepted them prior to the ban, but 13.9% did so after the ban. In this same time period, the proportion of vendors shipping via the US Postal Service rose from 83.5% in the overall sample to 92.7%; among the top 50, it went from 90% to 94.4% of vendors.

### ICV Traffic Analysis

**Figure 3:**
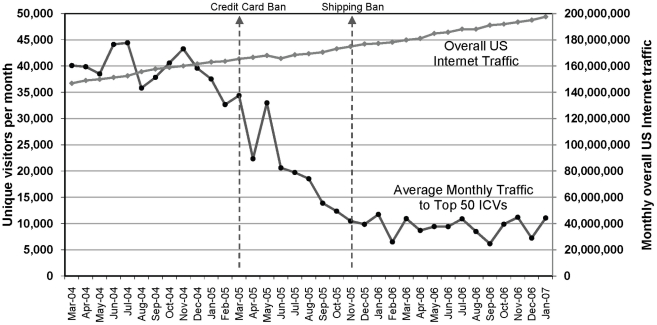
Visitor traffic 2004–2007 for 50 most popular Internet cigarette vendor websites. Average number of unique visitors from March 2004 to January 2007 for the 50 Internet cigarette vendor websites which were most popular in January 2005. Not all of the websites which were most popular in January 2005 were in business in March 2004. Sites were counted as missing data until they started receiving traffic.

**Figure 4:**
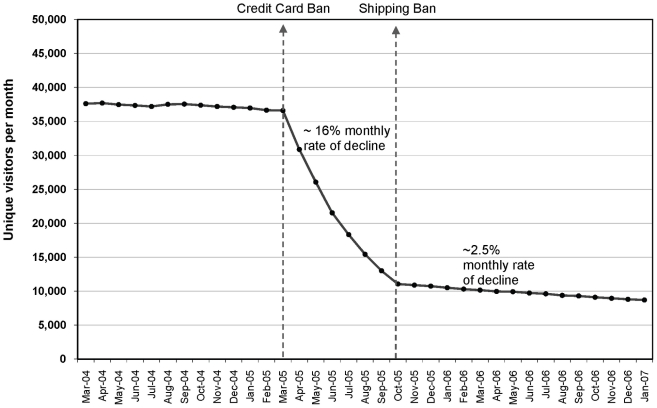
Visitor traffic for 50 most popular Internet cigarette vendor websites, adjusted for Internet traffic growth. Average predicted traffic for the 50 most popular Internet cigarette vendor websites, adjusted for Internet traffic growth (March 2004 to January 2007).

The results of the traffic analysis showed a substantial drop in traffic to ICV websites following the credit card and shipping bans. After adjusting for the increase over time in overall US Internet traffic, the analysis found a 3.5 fold decline in average Internet traffic to the top 50 ICV websites following the credit card ban in March 2005 (p = <0.0001). Following the shipping ban in October 2005, there was a further significant decline in ICV traffic (as compared to the period between the credit card and shipping bans). **Figure 3** displays the average traffic to the top 50 ICV websites from March 2004 to January 2007, and **Figure 4** illustrates the substantial declines in traffic following the bans after adjusting for changes in overall US internet traffic.

Piecewise regression analysis showed similar results. These was no detectable increase or decrease in traffic to the top 50 ICV websites 12 months prior to the credit card ban after adjusting for the overall Internet traffic. The rate of monthly ICV traffic declined drastically at a average monthly rate of 16% between the credit card ban in March 2005 and the shipping ban in October 2005 (p = <0.0001) (see **Figure 4**). Monthly traffic reached the peak average predicted count of approximately 36,000 unique visitors per site before the credit card ban, and declined to approximately 11,000 unique visitors per site right before the shipping ban. There were an estimated 1.25 million fewer visits per month at the time of implementation of the shipping ban for all 50 sites combined. After the shipping ban went into effect in October 2005, the declining trend continued at a slower but significant rate of a 2.5% average monthly traffic decline (p = <0.0001). The post-shipping ban decline in terms of predicted average number of visitors per month was approximately 3,000 (traffic declined from 11,000 to 8,000 unique visitors per month).

We also examined the possibility that one or a few sites going out of business drove the overall traffic decline. However, we found that this was unlikely to be the case, as more than a few went out of business, and traffic declined at nearly all sites following the bans, even for those that stayed open. By 1/06, 19 of the 50 vendors in the analysis had gone out of business, and by 1/07, 30 of the 50 vendors had gone out of business.

## Discussion

In the present study, the average monthly number of unique visitors to the most popular websites selling cigarettes declined by over three fold after the credit card ban and dropped even more after the shipping ban, albeit at a lower rate. In addition, the Websites quickly changed their sales practices and were less likely to offer the banned payment and shipping options and more likely to offer the few remaining payment and shipping options not covered by the agreements. Although the proportion of Web sites not in business after the agreements increased, there was growth in the number of new Internet vendors. To our knowledge, this is the first scientific study to examine the impact of these agreements. Our results suggest that the agreements appear to be achieving their intended goal of making it more inconvenient for potential buyers to buy cigarettes online, which has led to reduced traffic. The agreements do not fully curtail Internet sales because money orders and checks can still be used for payment, and delivery by the US Postal Service was not banned. The agreements did not require age verification or tax collection and remittance, two of the key policy concerns raised by Internet tobacco sales.

On March 31, 2010, President Obama signed the Prevent All Cigarette Trafficking (PACT) Act of 2009 (Public Law 111–154).^49^ The PACT Act makes cigarettes nonmailable matter (with a few minor exceptions) and requires all Internet cigarette vendors to verify the age and identity of customers and to pay all applicable taxes. This includes paying taxes for the destination state, which in most cases is a higher tax than the originating state. Thus, the federal PACT Act strengthened the provisions in the voluntary credit card and shipping agreements. Taken together, the AG agreement with private carriers to stop accepting Internet tobacco shipments and the PACT Act's making tobacco nonmailable matter through the US Postal Service has sharply limited the shipment options for Internet vendors. However, lawful Internet vendors (i.e., ones that verify age and collect and remit taxes) can transport tobacco products using messenger services, such as 4SameDay Solutions hppt://4sameday.com and Freight Center http://freightcenter.com/. The AG agreement is also a voluntary one and could be renegotiated if Internet vendors agree to make lawful sales by complying with the PACT Act provisions. Finally, states have new powers under the PACT Act, including the ability to enforce this law against out-of-state sellers. States with high state cigarette excise taxes will have the greatest incentive to enforce the PACT Act to ensure that Internet tobacco sales, if they are continuing, are not leading to tax evasion.

The transgeographical nature of the Internet defies regulation because proprietors of websites such as ICVs are often selling from tribal lands, offshore locations, and duty free zones, which are notoriously hard to regulate. [5] Because these bans intervened with the companies that do business with the ICVs and not with the vendors themselves, it appears to have made a stronger impact on the Internet cigarette sales industry. Prior efforts by states to intervene directly with ICVs or to collect unpaid taxes from buyers have been cumbersome and have met with limited success. [5], [16]

This study had several limitations. The main outcome was Web site traffic, the number of unique visitors, and the main goal of the policy was to reduce sales. We did not obtain actual purchase data, which are available for purchase but cost prohibitive. We determined the top 50 cigarette sales sites at baseline, but we do not have data on whether these sites were also the top 50 sites at other measurement times. Thus it is possible that these vendors represent a lesser amount of sales traffic at the other measurement occasions. This study was based on advertised payment and shipping methods, which might differ from those offered when a purchase attempt is made. For instance, some vendors list a credit card symbol on their Web site, but when a buyer attempts to use a credit card, the website states that they do not accept them or that their credit card payment system is malfunctioning. In a separate study in preparation we conducted test purchases using the banned payment and shipping methods to assess whether they would be blocked. Finally, longitudinal surveys of smokers should assess whether fewer smokers report purchasing from the Internet after these policies. Future studies should document whether the promising gains made by these agreements will be maintained and strengthened by the federal PACT Act. A wide variety of products of dubious benefit or that harm the public health are distributed via the Internet including genetic tests and services, [18] illicit drugs, [19] prescription drugs sold without a valid prescription, [20] alcohol, [21] and firearms. Approaches to regulating the sale and online distribution of these products can be informed by successful strategies governing Internet tobacco sales.
